# Deaths from Chloroform

**Published:** 1880-02

**Authors:** 


Deaths from Chloroform.—In consequence of a number of deaths having recently resulted from the use of chloroform, it behooves the profession to look around for some more safe anæsthetic in surgical practice; and we feel called upon to advise its disuse as much as possible. Dr. Turnbull's article on the *Bromide of Ethyl*, in this number, would seem to point to it as the *desirable substitute* for this dangerous anæsthetic, and as such we would suggest its trial in cases calling for general anæsthesia.

